# Body fluid derived exosomes as a novel template for clinical diagnostics

**DOI:** 10.1186/1479-5876-9-86

**Published:** 2011-06-08

**Authors:** Sascha Keller, Johannes Ridinger, Anne-Kathleen Rupp, Johannes WG Janssen, Peter Altevogt

**Affiliations:** 1 Department for Human Genetics, University of Heidelberg, D-69120 Heidelberg, Germany; 2 Tumor Immunology Programme, D015, German Cancer Research Center, D-69120 Heidelberg, Germany

## Abstract

**Background:**

Exosomes are small membrane vesicles with a size of 40-100 nm that are released by different cell types from a late endosomal cellular compartment. They can be found in various body fluids including plasma, malignant ascites, urine, amniotic fluid and saliva. Exosomes contain proteins, miRNAs and mRNAs (exosome shuttle RNA, esRNA) that could serve as novel platform for diagnosis.

**Method:**

We isolated exosomes from amniotic fluid, saliva and urine by differential centrifugation on sucrose gradients. Marker proteins were identified by Western blot and FACS analysis after adsorption of exosomes to latex beads. We extracted esRNA from exosomes, carried out RT-PCR, and analyzed amplified products by restriction length polymorphism.

**Results:**

Exosomes were positive for the marker proteins CD24, CD9, Annexin-1 and Hsp70 and displayed the correct buoyant density and orientation of antigens. In sucrose gradients the exosomal fractions contained esRNA that could be isolated with sufficient quantity for further analysis. EsRNAs were protected in exosomes from enzymatic degradation. Amniotic fluid esRNA served as template for the typing of the CD24 single nucleotide polymorphism (rs52812045). It also allowed sex determination of the fetus based on the detection of the male specific ZFY gene product.

**Conclusions:**

Our data demonstrate that exosomes from body fluids carry esRNAs which can be analyzed and offers access to the transcriptome of the host organism. The exosomal lipid bilayer protects the genetic information from degradation. As the isolation of exosomes is a minimally invasive procedure, this technique opens new possibilities for diagnostics.

## Background

Exosomes are membrane vesicles with a size of 40-100 nm that are released from many different cell types in the body such as red blood cells, platelets, lymphocytes, dendritric cells and also tumor cells [1-3]. Exosomes are formed by invagination and budding from the limiting membrane of late endosomes [4,5]. They accumulate in cytosolic multivesicular bodies (MVBs) from where they are released by fusion with the plasma membrane [4,5]. The process of vesicle shedding is very active in proliferating cells, such as cancer cells [6]. Depending on the cellular origin, exosomes contain various cellular proteins that may be different from proteins that are normally located in the plasma membrane including MHC molecules, tetraspanins, adhesion molecules and metalloproteinases [1,2,7]. Recent work has shown that, in addition to functional proteins, exosomes carry mRNA as well as miRNAs [8,9]. In functional terms, exosomes are considered to represent a novel mechanism of intercellular communication. This can be brought about by uptake of exosomes by target cells or by triggering cell signalling via membrane receptors [8,10].

In addition to their biological role in cell-cell communication, exosomes have been considered as novel tools for early diagnosis [11,12]. Indeed, exosomes can be isolated from various body fluids such as breast milk, serum, plasma, malignant ascites, and urine [9,13-17]. We have recently shown that exosomes derived from the fetus can be isolated from amniotic fluid collected during routine amnioscentesis [18]. These exosomes were derived in part from the renal system of the fetus as they carried kidney markers and could be distinguished by buoyant density from maternal exosomes [18]. However, the content of the shuttled RNA (exosomal shuttle RNA = esRNA) of these exosomes and their usefulness for diagnosis have not been investigated.

In the present publication we investigated for the first time in a systematic fashion whether esRNA can be used for diagnostic purposes. First we demonstrate that esRNA copurifies with exosomal protein markers on sucrose gradients and that esRNA can be isolated from exosomes from amniotic fluid, urine and saliva. Using the CD24 SNP (rs52812045, at position 170 from the CD24 translation start site) as a model system, we show that individuals can be successfully typed using esRNA as template. We also show that esRNA from amniotic fluid can be used to determine the sex of the fetus. Although the selected experimental examples are presently performed by standard methods, the use of esRNA represents the proof of principle of a new method using exosomes.

## Methods

### Human samples

Analysis of biological samples was carried out under the approval of the ethics commission of the University of Heidelberg. Amniotic fluid was collected for routine amniocentesis and analyzed after removal of cells. Urine and saliva samples were collected from healthy donors (male and female). For the isolation of microvesicles body fluids were spun for 20 min at 300 × g to remove cells and 20 min at 10,000 × g to remove cellular debris. The vesicles were pelleted using a Beckmann ultracentrifuge at 100,000 × g. The vesicle pellet was taken up in SDS sample buffer for direct analysis or further processed by sucrose density centrifugation. Mean values of exosomal protein isolated from amniotic fluid were: 36 μg/ml (range: 12 - 78 μg/ml, n = 93) and urine 6 μg/ml (range: 1.6 - 13 μg/ml, n = 14).

### Chemicals and antibodies

The mAb to human CD24 (SWA11) was described [19]. The mAbs to HSP70, Annexin-1, CD9, and ADAM10 were from BD-Transduction (Heidelberg, Germany).

### Sucrose density gradient fractionation

Isolated microvesicles were loaded onto the top of a step gradient comprising layers of 2 M, 1.3 M, 1.16 M, 0.8 M, 0.5 M and 0.25 M sucrose as described [14]. The gradients were centrifuged for 2.5 h at 100,000 × g in a Beckman SW40 rotor. Twelve 1 ml fractions were collected from the top of the gradient. For protein analysis the fractions were precipitated by acetone as described [14]. For esRNA isolation the gradient fractions were diluted with PBS and the exosomes were pelleted at 100,000 × g for 2 h and dissolved in RLT buffer (Quiagen, Hilden). Samples were analyzed by SDS-PAGE and Western blotting or submitted to RT-PCR as described below.

### Biochemical analysis

SDS-PAGE under reducing conditions and transfer of proteins to an Immobilon membrane using semi-dry blotting has been described [14,19]. After blocking with 5% skim milk in Tris-buffered saline (TBS), the blots were developed with the respective primary antibody followed by peroxidase conjugated secondary antibody and ECL detection.

### FACS analysis

FACS analysis of isolated vesicles was done after adsorbing isolated vesicles to 4 μm (Surfactant-free) aldehyde-sulfate latex beads (Interfacial Dynamics Corp., Portland OR, USA) as described [20]. The staining of beads with mAbs has been described [15,20]. Stained beads were analyzed with a FACS Canto using FACS Diva software (Becton & Dickinson, Heidelberg, Germany).

### Quantitative RT-PCR

10 ng of total cDNA were analyzed in triplicates. CD24 and GAPDH specific primers for qPCR were designed with Primer 3 Plus and were produced by MWG Eurofines (Ebersberg, Germany). The PCR reaction was performed with the SYBRgreen mastermix (Applied Biosystems, Darmstadt, Germany) in an ABI 7300 analyzer. Primers used for determining mRNA expression levels were as follows: CD24 fwd 5'-TGC CTC GAC ACA CAT AAA CC-3', CD24 rev 5'-GTG ACC ATG CGA ACA AAA GA-3'; GAPDH fwd 5'-ACA CCC ACT CCT CCA CCT TT-3', GAPDH rev 5'-TGC TGT AGC CAA ATT CGT TG-3'. To compare and quantify different measurements a cellular cDNA was used as standard and the amount was calculated after amplification.

### RNA / DNA purification and cDNA synthesis

Microvesicles were resupended in 350 μl RLT buffer and the isolation of esRNA was done using the Qiagen Allprep DNA/RNA Mini Kit according to the manufacturers protocol. CDNA was synthesized using reverse transcriptase (Fermentas, St. Leon-Rot, Germany) according to the manufacturers protocol. The quality control of RNA was done using a microfluidic-based Agilent 2100 bioanalyzer (Agilent Technologies, Böblingen, Germany).

### PCR and Restriction Fragment Length Polymorphism (RFLP)

Amplification from genomic DNA contaminants was avoided by designing primers from exon junctions (ExPrimer, http://exprimer.ibab.ac.in/exprimer_html/userguide.html). The first CD24 PCR amplification was done by using forward primer (5'-TCT CCA AGC ACC CAG CAT-3') and reverse primer (5'-CCC AAG AGA ACA GCA ATA GC-3'). The PCR conditions were as follows: 94°C for 1 min, 58°C for 1 min and 72°C for 1 min for 35 cycles. For the second PCR amplification the following primers were used: forward primer (5'-CCA CGC AGA TTT ATT CCA-3') and reverse primer (5'-CAT CAT CTA GTC AAA CCT CTC A-3'). The RT-PCR conditions were as follows: 94°C for 1 min, 54°C for 1 min and 72°C for 30 sec for 40 cycles. The analysis of the single nucleotide polymorphism (CD24 Ala/Val) was characterized by digestion of the PCR products for 2 h at 37°C with FastDigest BstXI (Fermentas) following electrophoresis on 2% agarose gels. The digestion patterns were as follows: the CD24 A/A genotype shows a single undigested 382 bp fragment, the CD24 V/V genotype gives two products (275 bp + 107 bp) and the CD24 A/V heterozygous genotype generates three products (382 bp + 275 bp + 107 bp).

The amplification of GAPDH by nested RT-PCR was done using the outer forward primer (5'-GGT CGT ATT GGG CGC CTG GT-3') and the outer reverse primer (5'-TTG AGG GCA ATG CCA GCC CC-3') with the following PCR conditions: 94°C for 1 min, 67°C for 1 min and 72°C for 30 sec for 35 cycles. Inner PCR was done with the forward primer (5'-TGC TGG CGC TGA GTA CGT CG-3') and the reverse primer (5'-ACA GTT TCC CGG AGG GGC CA-3') using the PCR conditions 94°C for 1 min, 67°C for 1 min and 72°C for 30 sec for 40 cycles. All primers were obtained from Eurofins MWG Operon (Germany), RedTaq Mix (Sigma, Germany) was used for RT-PCR according to the manufacturers protocol.

## Results

### Human saliva, urine and amniotic fluid contain exosomes

**Figure 1:**
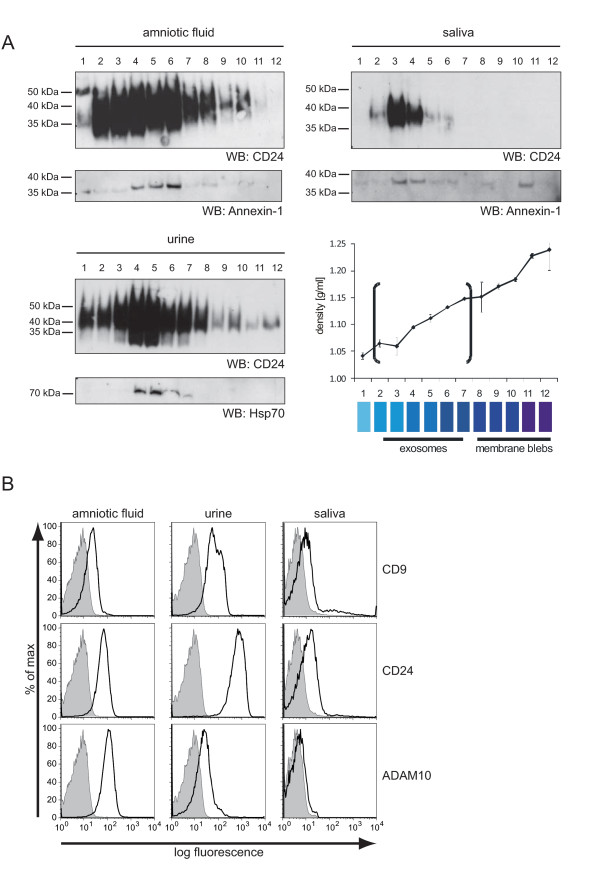
**Characterization of amniotic fluid, urine and saliva derived microvesicles**. (A) One representative example of microvesicles for amniotic fluid, saliva and urine was analyzed after sucrose density fractionation by Western blot analysis. (B) Isolated exosomes were adsorbed to latex beads and stained for the exosomal markers CD9, CD24 and ADAM10 followed by phycoerythrin-conjugated goat anti-mouse immunoglobulin G and FACS analysis. The negative control represents beads stained with the secondary antibody alone. The gray curve represents the autofluorescence of unstained beads. Note that the staining intensity is proportional to the amount of antigen on the exosomal surface.

We isolated exosomes by ultracentrifugation from the saliva of healthy donors. Likewise, exosomes were isolated from amniotic fluid collected at appr. week 16 of gestation for routine amniocentesis and urine as described before [18]. To demonstrate that the collected material represented exosomes, we determined the bouyant density by sucrose gradient centrifugation in combination with Western blot analysis. We found that the membrane vesicles between fractions 3-7 contained the established marker proteins CD24, Annexin-1 or Hsp70 and floated with the expected density of 1.08-1.14 g/ml (Figure 1A).

Exosomes are released from cells by fusion of MVBs with the plasma membrane and carry membrane antigens to the outside [1,2]. To determine the orientation of antigens, we immobilized exosomes onto latex beads and carried out FACS analysis. Vesicles were readily stained with antibodies to CD24, ADAM10 and CD9 (Figure 1B). In the saliva the detection of these markers was weaker compared to the other exosomes (Figure 1B).

### esRNA is protected from degradation

**Figure 2:**
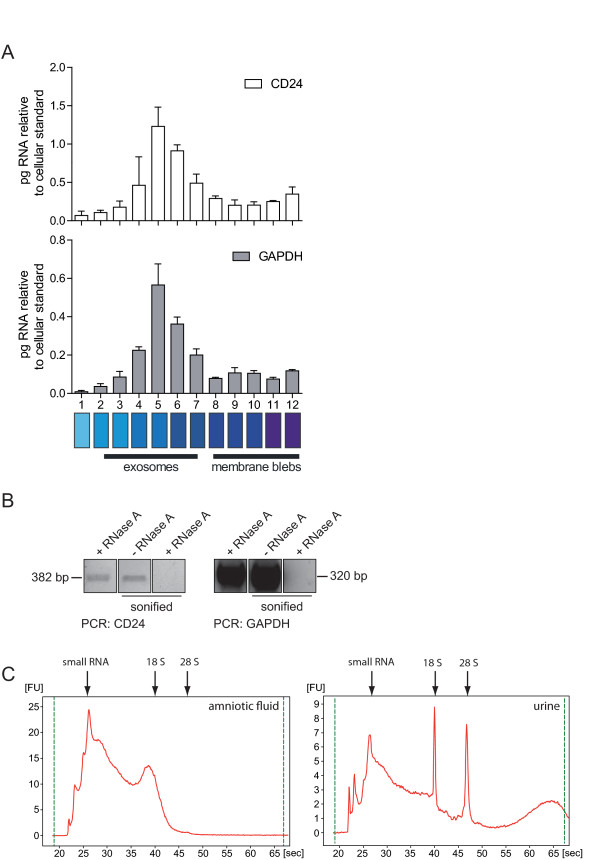
**Sucrose density analysis of urine derived microvesicles for RNA content**. (A) Urinary microvesicles were separated using sucrose density gradient centrifugation. The fractions were harvested, exosomal RNA was isolated and used for cDNA synthesis following RT-PCR analysis. (B) Isolated exosomes were incubated with RNase A alone or in combination with sonication to destroy the exosomal membranes. Isolated esRNA was analyzed by PCR. (C) Total RNA was isolated from amniotic fluid and urine exosomes and analyzed via an Agilent Bioanalyzer. The results show that exosomes contain variable amounts of 18 and 28S rRNAs as well as small and large RNAs.

Several studies have shown that esRNAs can be detected in exosomes [8,17,21]. Using urinary vesicles, we examined whether esRNA was indeed associated with exosomal fractions of the sucrose density gradient. For this purpose we collected the gradient fractions and subsequently isolated esRNA. CD24 and GAPDH message was detected by RT-PCR in fractions 4-7 of the gradient (Figure 2A) that co-localized with the exosomal marker proteins (see Figure 1A). To analyze whether the esRNA associated with exosomes was protected from degradation, we treated exosomes with RNase A and performed a qRT-PCR analysis (Figure 2B). We observed that esRNA was indeed protected from digestion as CD24 and GAPDH specific products could still be amplified (Figure 2B). However, the disruption of the exosomal membrane by sonication allowed the RNase to cleave esRNA and no RT-PCR product was detected (Figure 2B). These findings confirm and extend previous studies and suggest that only intact exosomes have RNase protecting abilities.

### The CD24 Ala/Val SNP can be detected in esRNA

The RNA content of amniotic fluid and urine exosomes was analyzed using a Bioanalyzer instrument, which showed that that both types of exosomes contain RNA, with little (urine) or no (amniotic fluid) ribosomal RNA (18S- and 28S-rRNA) (Figure 2C).

**Figure 3:**
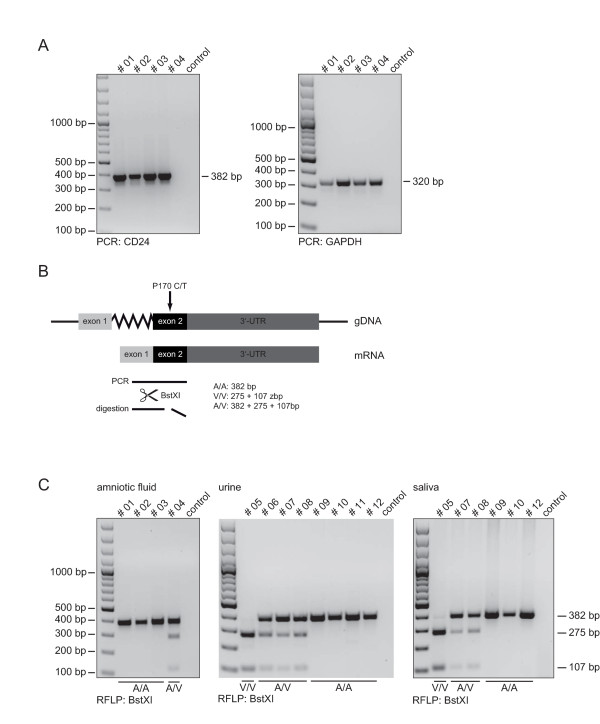
**CD24 SNP analysis using esRNA**. (A) Exosomal RNA was used as template for cDNA synthesis following PCR for CD24 and GAPDH. (B) Schematic overview of restriction fragement length polymorphism analysis. (C) CD24 PCR products were digested with BstXI for detection of the CD24 genotype. Note that samples from the same donor have the same number.

When four esRNA samples were subjected to RT-PCR analysis, both specific CD24 and GAPDH sequences could be amplified (Figure 3A).

The CD24 gene is crucial for the progression of autoimmune disease [22]. Two polymorphisms within the CD24 gene are known to modify disease risk and progression in multiple sclerosis (MS), systemic lupus erythematosus (SLE), giant cell arteritis, and in chronic hepatitis B [22]. A *C*>*T *SNP (rs52812045, at position 170 from the CD24 translation start site), is located in the putative GPI-anchor cleavage site (-1 position) of the CD24 protein, leading to a alanine (A) to valine (V) substitution [23]. The CD24 V/V genotype is associated with faster disease progression [22]. We selected this CD24 polymorphism as model system for our diagnostic readouts. The *C*>*T *nucleotide exchange results in the introduction of a BstXI cleavage site (Figure 3B). The amplified RT-PCR products were analyzed by RFPL after BstXI digestion. We could clearly identify the CD24 SNP (Figure 3C).

To examine whether SNP-typing could also be applied to other exosomal samples, we used urine and saliva exosome derived esRNAs as template. Indeed, identical results were obtained for urine as well as saliva exosomes (Figure 3C). As the samples were derived from the same donors, the results can be easily compared. We also verified the genotypes using genomic DNAs derived from blood leukocytes of all donors and found complete identity (data not shown).

### Fetal sex determination using amniotic fluid exosomes

**Figure 4:**
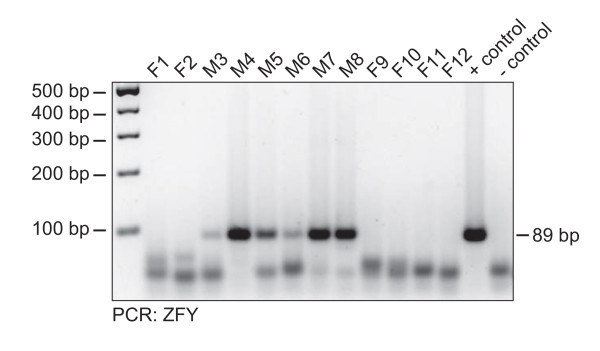
**Sex determination using amniotic fluid derived esRNA**. Twelve different amniotic fluids were analyzed for the gender of the fetus. Exosomal RNA was used as template for cDNA synthesis following gender specific PCR for ZFY. Note that bands running in the front of the gel represent unconsumed primers of the PCR reaction.

The sex-determining region of the human Y chromosome encodes a zinc finger protein ZFY that is important for fetal development [24]. Earlier studies reported that in late pregnancy fetal RNA can be detected in maternal plasma and ZFY mRNA can be used for sex determination of the fetus [25]. We adressed the question whether mRNA encoding ZFY was present in exosomes. We used mRNA from the male or female derived cell lines as controls. Exosomes from 12 amniotic fluids were analyzed by RT-PCR using ZFY specific primers in a blinded fashion. 6 of 12 samples revealed an often strong and unambiguous band of the expected size (Figure 4). Decoding of the sample revealed a 100% match with conventional cytogenetic analysis.

## Discussion

Microvesicles in body fluids are a heterogenous group of cell-released vesicles composed of exosomes, microparticles and apoptotic membrane blebs as its main representatives. They are mostly composed of proteins and lipids but also contain nucleic acids. In the present report we demonstrate that a recently discovered population of membrane vesicles termed exosomes, carry genetic information that can be used for diagnostic purposes. We demonstrate that i) esRNA of sufficient quantity can be extracted from body fluid exosomes, that ii) the genetic information is protected from degradation in exosomes, and that iii) in selected examples the esRNA can be used for the determination of SNPs in transcripts as well as for the detection of specific transcripts. We propose that the analysis of esRNA could provide new insights into the transcriptome of the body for example during disease or pregnancy.

For prenatal diagnostics fetal cells are often obtained by invasive procedures like amnioscentesis or chorion villus sampling. These methods constitute a risk of fetal misscarriage and injury and are therefore only offered to women with/at high-risk pregnancies. One of the most promising approaches is the use of cell-free nucleic acids in sera. Cell-free fetal DNA (cff DNA) was first discovered in 1997 in maternal plasma and serum of pregnant women and offers an excellent posibility as starting material for non-invasive prenatal diagnosis [26,27]. The majority of cell free DNA is of maternal origin, only 3-6% of circulating cell-free DNA is of fetal origin [26]. This limits further analysis of cff DNA to fetal targets differing from the maternal ones. Additionally, cell-free fetal DNA and RNA have been isolated from other body fluids e.g. maternal plasma [27], amniotic fluid [28], and cerebrospinal fluid [29]. Although not tested at that time, it is quite likely that these nucleic acids are associated with microvesicles which could explain their relative stability in the nuclease-rich environment of body fluids. The enrichment of fetal derived exosomes by marker proteins is a big challenge and would allow the discrimination between maternal and fetal cell-free nucleic acids.

Microparticles, i.e. exosomes are also present in serum, pleural effusions and ascites of cancer patients [9,14-16]. As stated above, these exosomes most likely represent a mixture derived from various cell types. Recently, we have shown that exosomes derived from the tumor can be distinguished from normal cell exosomes by marker expression [30]. Exosomes in the ascites derived from ovarian cancer carried the marker set EpCAM, CD24 and CD9 that appear to exist on a common exosome type [30]. In the present study we used for the analysis of amniotic fluid, urine and saliva exosomes other exosomal marker proteins such as Annexin-1, CD24, HSP-70 or ADAM10. It should be pointed out that at presence there is no evidence that these markers are shared by all exosomes.

An important feature is that, just like cells, exosomes can be isolated by antibodies and MACS procedures. Thus, mAb to membrane proteins overexpressed in tumors such as CD24 or EpCAM can be used to enrich tumor derived exosomes [30,31]. This technique is not only limited to the body fluid surrounding the tumor, as exosomes can become detectable in the serum and therefore allows minimal invasive collection methods [15]. The miRNA profiling of ovarian malignant ascites derived exosomes revealed unique expression signatures derived from the tumor [31]. Exosomes from glioblastoma patients expressed esRNA for a truncated and oncogenic form of the epidermal growth factor receptor, known as EGFRvIII that can be transferred via exosomes to neighbouring cells [32]. Thus, it is possible that exosomes derived from the tumor can serve as messengers (for their diagnosis) and mediators of tumor progression [33].

Although knowledge about the secretion from MVBs and the requirements for protein sorting into exosomes is growing, it is presently not known how genetic information is recruited into exosomes. An important question is whether the esRNA and miRNA content of exosomes is representative for the cell of origin. Valadi et al showed that microarray assessments of esRNA from mouse and human mast cell lines revealed the presence of mRNA from approximately 1,300 genes, many of which were not present in the cytoplasm of the donor cell [21]. Another study reported that miRNA from ovarian tumor cells and exosomes from the same patients were positive for 218 of 467 mature miRNAs analyzed. The levels of only 8 specific microRNAs were similar between cellular and exosomal miRNAs [31]. Further studies are needed to address this important question.

## Conclusions

The results presented in this report suggest that esRNAs could give new insights into the transcriptome. It provides an explanation why nucleic acids were detected in body fluids. We are aware of the fact that both CD24 genotyping and fetal sex determination are presently done very efficiently by standard methods. But the use of esRNA for further diagnostics is the proof of principle of a new method using exosomes. This could be of great importance when cellular material is not accessible.

## Abbreviations

esRNA: exosomal shuttle RNA; mAb: monoclonal antibody; MVB: multivesicular bodies; SNP: single nucleotide polymorphism; RFLP: restriction fragment length polymorphism

## Authors' contributions

SK, JR and AR performed experiments. JJ was instrumental in collecting and provided amniotic fluids. PA is the corresponding author of this paper and was critical for the study design and writing of the manuscript. All authors have read and approved the final manuscript.

## Competing interests

The authors declare that they have no competing interests.
